# The Safety and Effectiveness of Decapeptide in Patients With Vitiligo: A Real-World Study

**DOI:** 10.7759/cureus.41418

**Published:** 2023-07-05

**Authors:** Aseem Sharma, Imran Majid, Hari K Kumar, Pravin Banodkar, Madhulika Mhatre, Bhagyashree Mohod, Ashok Jaiswal

**Affiliations:** 1 Department of Dermatology, Skin Saga Centre for Dermatology, Mumbai, IND; 2 Department of Dermatology, Cutis Institute of Dermatology, Srinagar, IND; 3 Department of Dermatology, Rajarajeswari Medical College, Bangalore, IND; 4 Department of Dermatology, Saifee Hospital, Mumbai, IND; 5 Department of Medical Affairs, Zydus Healthcare Limited, Mumbai, IND

**Keywords:** vitiligo, re-pigmentation, melanocyte, decapeptide, basic fibroblast growth factor

## Abstract

Background

Vitiligo, a chronic autoimmune depigmenting skin disease, affects a significant portion of the global population. One of the topical treatment options for vitiligo is basic fibroblast growth factor (bFGF)-related decapeptide (bFGFrP) 0.1% solution. This study aimed to assess the real-world effectiveness and safety of decapeptide in treating vitiligo.

Methods

This retrospective analysis utilized data collected from routine clinical practice in the management of vitiligo, focusing on patients treated with topical decapeptide lotion (Melgain™, manufactured by Zydus Healthcare Ltd., Ahmedabad, India). The primary outcome measures included the extent of re-pigmentation (EOR) and the grade of re-pigmentation (GOR) assessed at each follow-up visit.

Results

The analysis included data from 65 patients (24 males and 41 females) with an average age of 30.83 years. Segmental vitiligo was present in 52.31% of cases, with the face being the most commonly affected site. Among the patients, 33 received decapeptide as monotherapy, while 32 received decapeptide alongside adjuvant drug/phototherapy. The mean duration of treatment was five months. The first, second, and final follow-ups were observed to be at a mean of 45 days, two months, and five months, respectively.

During the second and final follow-up, a significant response (>75% re-pigmentation) was observed in 12% (eight) and 71% (46) of the patients. A mild response (<50% re-pigmentation) was noted in 45% (29) of the patients during the first follow-up visit, 15% (10) during the second follow-up visit, and 6% (four) during the final follow-up visit. Grade 6 and 7 re-pigmentation occurred in a higher number of patients at the final visit, indicating treatment effectiveness. Overall, nearly all patients (96.92%) reported excellent tolerability of the decapeptide lotion based on the global assessment of tolerability.

Conclusion

This real-world study demonstrates that decapeptide promotes re-pigmentation and improves patient outcomes in vitiligo. Both decapeptide regimens, as monotherapy or in combination with other therapies, were effective and well tolerated by most patients. Thus, decapeptide represents a safe and effective therapeutic option for vitiligo treatment.

## Introduction

Vitiligo is a chronic autoimmune depigmenting skin disease characterized by the loss of epidermal melanocytes, which affects 0.5%‒1% of the world's population without a gender and racial disparity. Vitiligo heavily impacts the quality of life (QoL), especially when visible areas are involved [1]. India has recorded a prevalence ranging from 0.5% to 4%, rising up to 8% in Gujarat and Rajasthan [2,3]. Vitiligo is characterized by generalized depigmentation of the skin and mucosa, related to genetic factors, self-destruction of melanocytes, cytokines, autoimmunity, and oxidative stress. Although the exact pathogenesis is yet to be determined, the deficiency of the basic fibroblast growth factor (bFGF) has a significant role in the pathophysiology of vitiligo. The onset of vitiligo can be at any age, and the disease has a grave psychological impact mainly due to the social stigma attached to this condition. Many studies also have demonstrated the association of vitiligo with other autoimmune diseases, such as alopecia areata, rheumatoid arthritis, type 2 diabetes mellitus, Addison's disease, pernicious anemia, systemic lupus erythematosus, and psoriasis. Vitiligo treatment involves phototherapy, surgical procedures, immunosuppressive agents (methotrexate, azathioprine, cyclosporine, Janus kinase inhibitors, and biologics), and topical therapies, such as glucocorticoids, calcineurin inhibitors, vitamin D, and bFGF-related decapeptide (bFGFrP) [4].

Treatment options are limited and are mainly based on immunosuppressive agents and the use of ultraviolet (UV) light. The current treatments for vitiligo remain suboptimal, which may not be equally effective in all vitiligo patients. In clinical practice, treatment modalities are chosen in the individual patient, based on disease severity, disease activity (stable versus progressive disease), patient preference (including cost and accessibility), and response evaluation [4].

It is well established that patients with vitiligo have decreased bFGF mRNA expression. The loss of bFGF is implicated in pigment loss, and hence, it can be an ideal treatment [5]. Vitiligo is a depigmentation disorder characterized by the loss of functional melanocytes of the skin epidermis, and bFGF facilitates melanocyte migration via various signalling pathways [6].

bFGF-related decapeptide 0.1% solution is a topical treatment modality for vitiligo. Based on the studies, growth factors for melanocytes such as bFGF or other growth factors may be involved in re-pigmentation [7-10]. bFGF-related decapeptide helps in re-pigmentation by stimulating the growth and migration of melanocytes. The rationale for decapeptide in the treatment of vitiligo is based on the melanocyte growth factor deprivation theory, and the clinical evidence generated in the Indian clinical trials supports decapeptide as a drug for the management of vitiligo [11]. Based on the clinical trials, decapeptide was approved in India for the treatment of vitiligo [10].

There is no published real-world clinical data available to substantiate the benefits of decapeptide in the treatment of vitiligo. Therefore, we retrospectively analyzed 65 patients with vitiligo who received decapeptide lotion to confirm the effectiveness and safety of this therapy in the real-world scenario.

## Materials and methods

Study design

This was a retrospective analysis of the data collected from multiple centers in different parts of India. The protocol and design of the study were approved by the Suraksha Ethics Committee, Thane, India (dated 03/06/2023, institutional ethics committee (IEC) registration number ECR/644/Inst/MH/2014/RR-20).

Setting and participants

The data of the patients with vitiligo who had visited the dermatology outpatient department and received decapeptide as monotherapy or along with other concomitant treatments for vitiligo were retrieved from medical records. The data of the patients that were prescribed topical decapeptide lotion (Melgain™, manufactured by Zydus Healthcare Ltd., Ahmedabad, India) for the treatment of vitiligo (both old cases and newly diagnosed) were included in the analysis. Pregnant or lactating females and those with an allergy to decapeptide were excluded from the analysis. The data of the included patients were collected in a case record form. Since this was a real-world study, concomitant medication for vitiligo treatment was recorded and analyzed. The data from three follow-up visits posttreatment initiation were captured.

Effectiveness and safety

The primary objective of this study was a parameter to evaluate the effectiveness of treatment based on the extent of re-pigmentation (EOR), categorized as a marked response (>75%), moderate response (50%-75%), mild response (<50%), or no response. The grade of re-pigmentation (GOR), ranging from 1 to 7 (with grade 1 indicating no change in the depigmentation area and grade 7 representing complete re-pigmentation), was recorded at each follow-up visit. Patient global assessment (PGA) was conducted at the end of treatment, capturing the patient's response on a scale ranging from no change (zero improvement) to complete improvement (100% improvement). Safety was assessed by monitoring the incidence of any adverse events and obtaining a global assessment of tolerability.

Statistical analysis

The data were analyzed for demographics, safety, and effectiveness. The data are presented as mean ± standard deviation (SD) or number (percentage). The data (observations) for the percentage of re-pigmentation were on ordinal scale (gradation). All statistical analyses were performed using the Statistical Package for Social Sciences (SPSS) version 26.0 statistical software (IBM SPSS Statistics, Armonk, NY).

## Results

The data on 65 vitiligo patients (24 males and 41 females) that received decapeptide lotion were retrospectively analyzed (Table 1).

**Table 1:** Demographic details of the included patients with vitiligo

Parameter	N (total = 65)	%
Age		
<12 years	10	15.38%
13-20	7	10.76%
21-40	31	47.69%
41-60	16	24.61%
>60	1	1.53%
Gender		
Male	24	37%
Female	41	63%
Family history		
No	59	90.77%
Yes	6	9.23%
Duration of the disease		
<1 year	24	36.92%
1-3 years	13	20.00%
3-5 years	8	12.31%
>5 years	20	30.77%
Presence of comorbidity		
No	58	89.23%
Yes	7	10.77%
Type of vitiligo		
Segmental	34	52.31%
Non-segmental	31	47.69%
Number of lesions		
1	7	10.77%
2	9	13.85%
3	8	12.31%
4	12	18.46%
5	3	4.62%
>5	26	40.00%
Site of vitiligo		
Arms	14	21.54%
Face	23	35.38%
Neck	5	7.69%
Legs	7	10.77%
Chest	3	4.62%
Trunk	3	4.62%
Others	10	15.38%
Monotherapy with decapeptide	33	50.77%
Decapeptide combination therapy with:	32	49.23%
Tacrolimus	6	
Phototherapy	8	
Steroid	2	
Tacrolimus + phototherapy	4	
Tacrolimus + adjuvants (other drugs)	3	
Other combinations	11	

The mean age ± SD was 30.83 ± 16.42 years. Most of the patients belonged to the age group of 21-40 years. A family history of vitiligo was observed in six cases (9.23%). About 36.92% of the patients had vitiligo for less than one year, and 30.77% of the patients had vitiligo for more than five years. Only seven patients (10.77%) had a comorbidity. Segmental vitiligo was observed in 52.31% of the cases, while the remaining 47.69% of the patients had non-segmental vitiligo. Out of 65 patients, 26 (40%) had more than five vitiligo lesions. In 35.38% of the patients, the face was the most common site for vitiligo lesions.

Out of 65 patients, 33 (50.77%) had received decapeptide monotherapy, while the remaining patients (32) were on combination therapy. The mean duration of treatment was five months. The first, second, and final follow-ups were observed to be at a mean of 45 days, two months, and five months, respectively.

Efficacy

**Figure 1:**
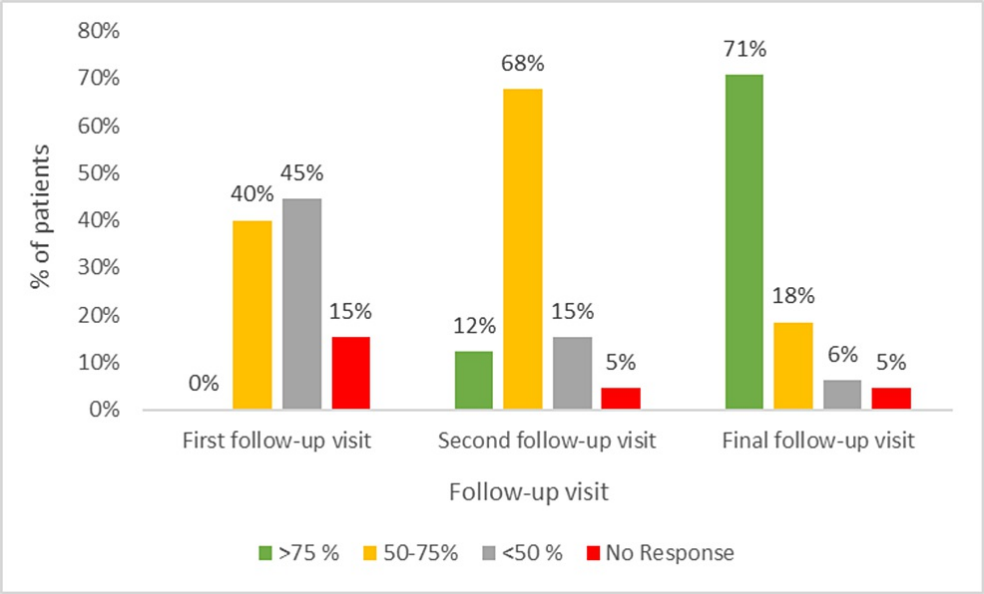
Percentage of re-pigmentation in the patients at different follow-up visits

Response to treatment improved over time, and at the first follow-up visit, 40% (26) of the patients showed moderate response (50%-75% re-pigmentation), and 45% (29) of the patients showed mild response (<50% re-pigmentation). During the second follow-up visit, a marked response (>75% re-pigmentation) was achieved in 12% (eight) of the patients, 68% (44) of the patients showed moderate response, and 15% (10) had mild response (Figure 1).

**Figure 2:**
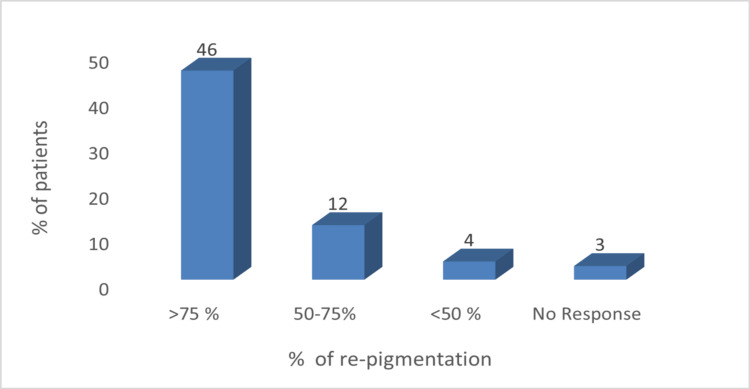
Number of patients achieving different percentages of re-pigmentation

During the final follow-up visit, 71% (46) of the patients achieved marked response, 18% (12) had moderate response, and only 6% (four) of the patients showed mild response (Figure 2).

Only 5% (three) of the patients did not have re-pigmentation at the end of treatment.

Grade 6 and 7 re-pigmentation was seen in a greater number of patients during the final visit compared to the first follow-up visit, indicating treatment effectiveness. The grade of re-pigmentation improved overall at each follow-up visit (Table 2).

**Table 2:** Grade of re-pigmentation during each follow-up visit

Grade	Definition of grade	First follow-up visit, n (%)	Second follow-up visit, n (%)	Final follow-up visit, n (%)
Grade 1	No change in the depigmentation area	10 (15%)	2 (3%)	2 (3%)
Grade 2	Specks of re-pigmentation or concavity of margins	31 (48%)	7 (11%)	4 (6%)
Grade 3	The area of re-pigmentation is less than the depigmentation area	20 (31%)	21 (32%)	13 (20%)
Grade 4	The area of re-pigmentation is equal to the depigmentation area	2 (3%)	16 (25%)	7 (11%)
Grade 5	The area of re-pigmentation is more than the residual	2 (3%)	15 (23%)	9 (14%)
Grade 6	Some specks of depigmentation left	0 (0%)	4 (6%)	26 (40%)
Grade 7	Complete re-pigmentation	0 (0%)	0 (0%)	4 (6%)

**Figure 3:**
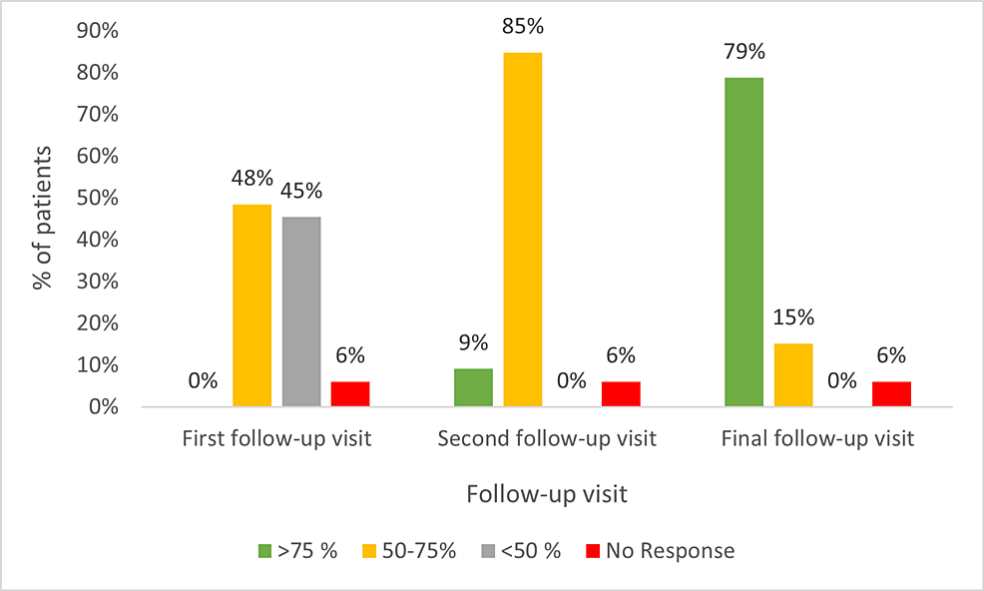
Percentage of re-pigmentation in the patients on monotherapy

Decapeptide monotherapy (n = 33) led to a marked response (>75% re-pigmentation) in 9% (three) and 79% (26) of the patients during the second and final follow-up visits, respectively. A moderate response was observed in 48% (16), 85% (28), and 15% (five) of the patients during the first, second, and final follow-up visits, respectively (Figure 3).

**Figure 4:**
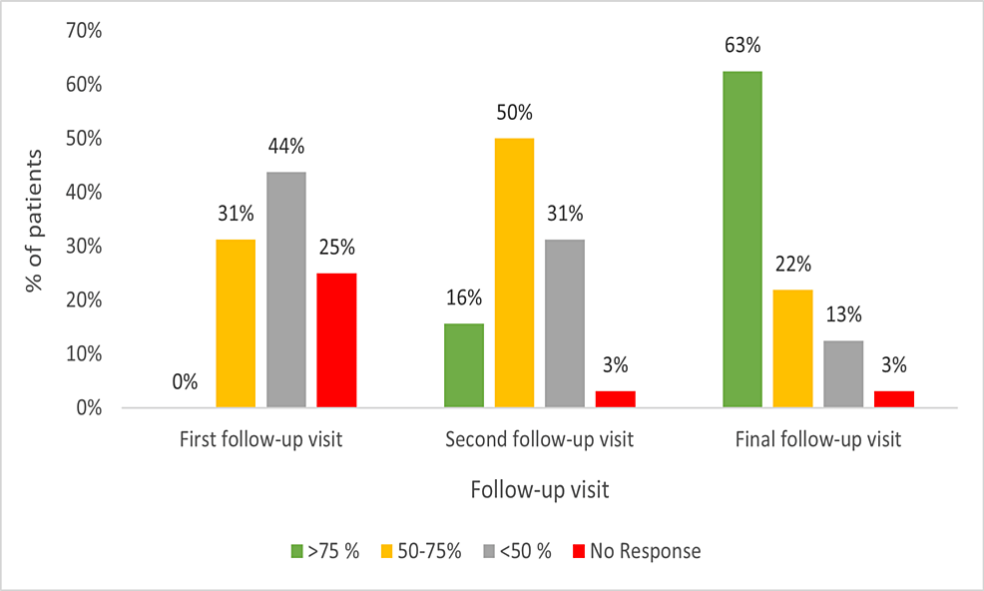
Percentage of re-pigmentation in the patients on combination therapy

Grade 6 and 7 re-pigmentation was observed in 50% of the monotherapy-treated patients at the end of treatment. About 16% (five) and 63% (20) of the patients on combination treatment (32) had >75% re-pigmentation during the second and final follow-up visits. A moderate response was observed in 31% (10), 50% (16), and 22% (seven) of the patients during the first, second, and final follow-up visits. Grade 6 and 7 re-pigmentation was observed in 43.75% (12) of the patients that received combination therapy (Figure 4).

**Figure 5:**
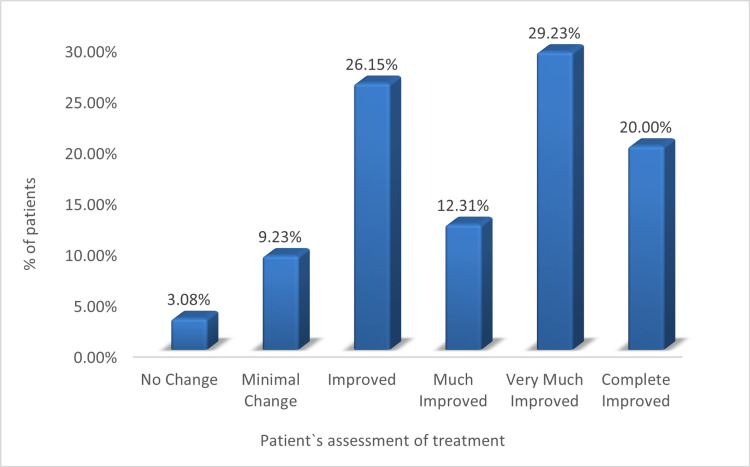
Patient global assessment of treatment effectiveness

As per patient global assessment, more than 87% of the patients felt that their vitiligo improved after decapeptide therapy. Complete improvement was observed in 20% (13) of the patients (Figure 5).

Tolerability and safety

**Figure 6:**
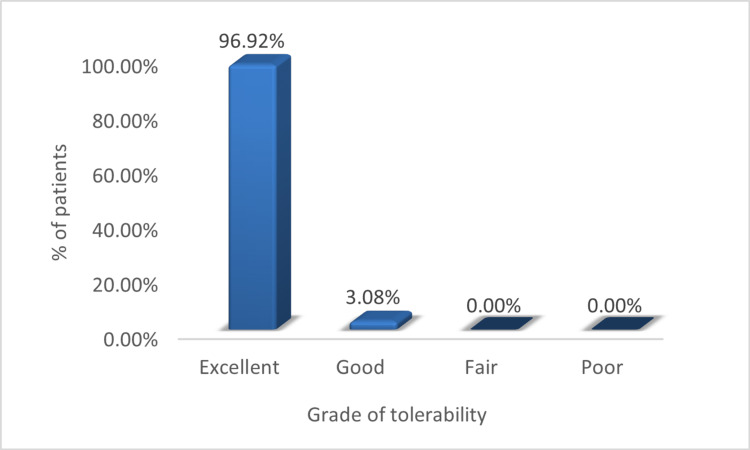
Results of the global assessment of tolerability after decapeptide treatment

The treatment with decapeptide lotion was well tolerated. No major adverse reactions were observed. Only one patient experienced erythema and burning sensation on application that subsided soon and did not lead to treatment discontinuation or the requirement of additional management. Almost all the patients (96.92%) had excellent tolerability with decapeptide lotion (Figure 6).

**Figure 7:**
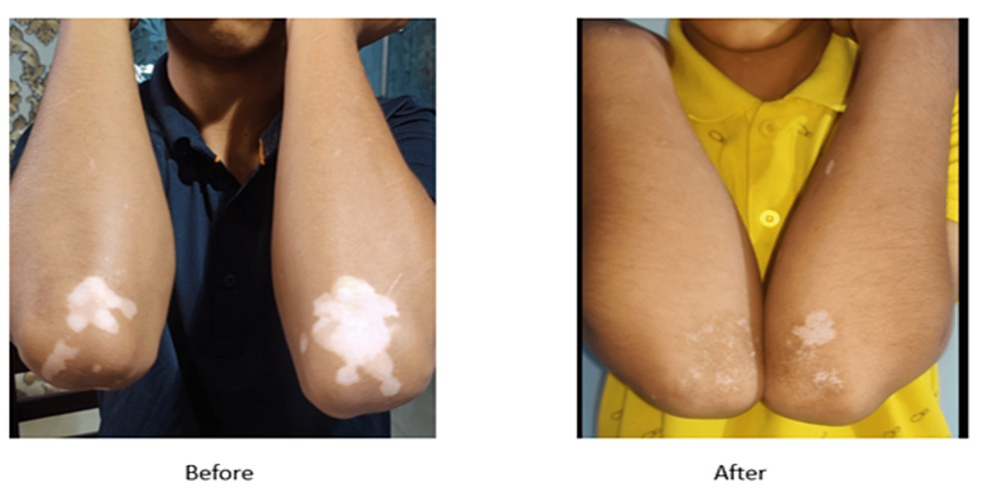
Before and after images of vitiligo lesion in a 14-year-old male patient that received topical decapeptide lotion plus topical mometasone

**Figure 8:**
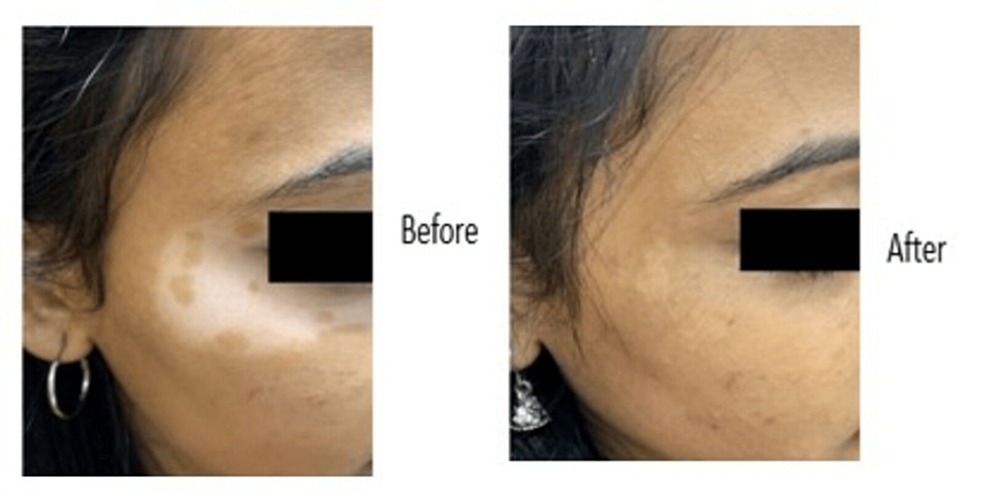
Before and after images of vitiligo lesion in a 23-year-old female patient that received the combination of topical decapeptide lotion, topical tacrolimus, and phototherapy, who was previously on a combination of tacrolimus and phototherapy

The before and after images of the vitiligo lesion of the two patients are shown in Figures 7-8.

## Discussion

The psychosocial impact of vitiligo on patients emphasizes the need for increased focus on its management. The selection of an effective and well-tolerated treatment for vitiligo remains a prominent subject of interest in the field of dermatology. Active peptides, such as decapeptide derived from bFGF, have emerged as therapeutic agents for vitiligo management, as they stimulate melanogenesis, the process of pigment production in the skin [9,12]. Randomized clinical trials in India have confirmed the efficacy and safety of decapeptide in the Indian population [10,11].

The findings from this study demonstrate significant improvement in re-pigmentation among 98% of the patients, with 71% of them achieving a marked response (>75% re-pigmentation) during the final follow-up. Only a small subset of three patients did not exhibit a response, which may be attributed to factors such as noncompliance with treatment or the severity of their vitiligo. The data indicate that decapeptide monotherapy effectively treats both segmental and non-segmental types of vitiligo. Moreover, a marked response (>75% re-pigmentation) was observed in 79% of the patients during the final follow-up, and approximately 50% of the patients achieved grade 6 and 7 re-pigmentation levels. In the study by Subhashini et al., decapeptide 0.1% was more effective than betamethasone valerate 0.1% [13].

In an Indian-based study by Girish et al., bFGF decapeptide lotion was prescribed to 54.1% of the patients [14]. Decapeptide has been shown to act synergistically to give better results in terms of better re-pigmentation and faster response rates when combined with other treatment modalities for vitiligo [15]. In the current study, both forms of decapeptide regimens (monotherapy and combination) were effective in vitiligo management. Tacrolimus and phototherapy-based regimens were commonly prescribed along with topical decapeptide for the treatment of vitiligo.

Improvement in the extent of re-pigmentation was seen in this study. About 89.2% of the patients achieved greater than 50% re-pigmentation after decapeptide treatment. This rate is higher in this real-world study compared to 66.7% in another study by Parsad et al., involving a combination treatment with decapeptide and tacrolimus for 12 months [11]. In another study conducted by Nayak et al., 61.8% of the patients achieved significant EOR of >50% after bFGF and oral psoralen plus ultraviolet A (PUVA) combination therapy [16]. A study by Ramaiah and Madhava showed that topical decapeptide treatment for 12 weeks resulted in the visible reduction of wrinkles and fine lines on the macules in the patients with stable non-segmental vitiligo [17]. The combination of narrowband ultraviolet B (NBUVB) light and decapeptide for three months was superior to NBUVB alone in re-pigmenting vitiligo macules [10].

In the current study, decapeptide had a favorable safety profile and was well tolerated without any major side effects, similar to other studies [10,18]. Since this was a real-world evidence study, there are certain limitations such as small sample size and open-label study design. Conducting a multicentric, randomized study would help in assessing the efficacy and safety in a larger population.

## Conclusions

This real-world study demonstrated that the utilization of topical decapeptide therapy resulted in enhanced re-pigmentation and improved patient outcomes for individuals with both segmental and non-segmental vitiligo. Both the monotherapy and concomitant therapy approaches using decapeptide yielded positive effects on the percentage and grade of re-pigmentation. Overall, the topical decapeptide regimen proved to be an effective and safe treatment option for segmental and non-segmental vitiligo.
